# Pulmonary Artery Catheter (PAC) Accuracy and Efficacy Compared with Flow Probe and Transcutaneous Doppler (USCOM): An Ovine Cardiac Output Validation

**DOI:** 10.1155/2012/621496

**Published:** 2012-05-09

**Authors:** Robert A. Phillips, Sally G. Hood, Beverley M. Jacobson, Malcolm J. West, Li Wan, Clive N. May

**Affiliations:** ^1^School of Medicine, The University of Queensland, Brisbane QLD 3010, Australia; ^2^USCOM Ltd., Department of Clinical Science, Sydney NSW 3010, Australia; ^3^Howard Florey Institute, University of Melbourne, Parkville VIC 3010, Australia; ^4^Department of Pharmacology, University of Melbourne, Parkville VIC 3010, Australia

## Abstract

*Background*. The pulmonary artery catheter (PAC) is an accepted clinical method of measuring cardiac output (CO) despite no prior validation. The ultrasonic cardiac output monitor (USCOM) is a noninvasive alternative to PAC using Doppler ultrasound (CW). We compared PAC and USCOM CO measurements against a gold standard, the aortic flow probe (FP), in sheep at varying outputs. *Methods*. Ten conscious sheep, with implanted FPs, had measurements of CO by FP, USCOM, and PAC, at rest and during intervention with inotropes and vasopressors. *Results*. CO measurements by FP, PAC, and USCOM were 4.0 ± 1.2 L/min, 4.8 ± 1.5 L/min, and 4.0 ± 1.4 L/min, respectively, (*n* = 280, range 1.9 L/min to 11.7 L/min). Percentage bias and precision between FP and PAC, and FP and USCOM was −17 and 47%, and 1 and 36%, respectively. PAC under-measured Dobutamine-induced CO changes by 20% (relative 66%) compared with FP, while USCOM measures varied from FP by 3% (relative 10%). PAC reliably detected −30% but not +40% CO changes, as measured by receiver operating characteristic area under the curve (AUC), while USCOM reliably detected ±5% changes in CO (AUC > 0.70). *Conclusions*. PAC demonstrated poor accuracy and sensitivity as a measure of CO. USCOM provided equivalent measurements to FP across a sixfold range of outputs, reliably detecting ±5% changes.

## 1. Background

Since its introduction in 1970 [1], the Swan Ganz pulmonary artery catheter (PAC), using the thermodilution method (TD), has been accepted as a gold standard for the clinical measurement of cardiac output (CO). The PAC has been used to evaluate and guide clinical care, to develop our understanding of physiology and pathophysiology, and as a reference standard for evaluation of novel CO measurement methods. Despite this widespread application over the last 40 years, it remains essentially without validation and without clinical outcomes benefit [2–5]. Explanations for the absence of PAC effectiveness may be the uncertain accuracy of the method [6–12]. Additionally, PAC TD, using either bolus injections or continuous thermometric monitoring [1, 13, 14], is invasive, with associated patient risks [15–18], and is costly [19].

Given the importance of the circulation in clinical practice, the frequency of clinical interventions, and the limitations of PAC, there is a need for an improved CO measurement and monitoring method. CO_2_ partial re-breathing [20], breath-to-breath pulmonary blood flow measures [21], arterial pulse pressure analysis [22], and transesophageal Doppler [23] have also been used to measure CO. However, these alternatives have limitations which have precluded significant adoption. A noninvasive, accurate, and effective alternative to PAC may improve clinical care and contribute to our understanding of circulation.

The Ultrasonic Cardiac Output Monitor, (USCOM) (USCOM 1A, Uscom Ltd., Sydney, NSW, Australia), is a noninvasive, morphometrically calibrated, continuous wave (CW) Doppler ultrasound device which provides an instantaneous, beat-to-beat measure of right- and left-sided CO. CW Doppler is a widely adopted clinical tool with an accuracy measured by Doppler string phantoms of ±2.3% [24]. USCOM has been compared positively with flow probes in animals [25], with echocardiography from 0.12 L/min in neonates [26], in externally driven artificial hearts in orthotopic transplantation [27], with PAC in the postcardiac surgical setting [28–31], and with PAC from 2.14 to 18.7 L/min in liver transplantation [32, 33]. It has acceptable reproducibility in adults and children [34–36], and has been recommended as an alternative to PAC [37].

The ultrasonic transit-time flow probe (FP) is considered a true gold standard method for measuring beat-to-beat CO with an accuracy of ±1 to 2% [38] but is limited to animal studies as it requires surgical implantation.

This study compared the two clinical methods of CO measurement, PAC and USCOM, with measures from an implanted FP in conscious sheep to evaluate the relative accuracy and sensitivities of the methods across a range of outputs at baseline and during pharmacologic interventions.

## 2. Methods

### 2.1. Study Design and Data

The study was approved by the Animal Experimentation Ethics Committee of the Howard Florey Institute. Prior to experiment, 10 adult Merino ewes were anaesthetised with intravenous (i.v.) sodium thiopental (15 mg/kg), and, following intubation, anesthesia was maintained with 1.5–2.0% isoflurane in oxygen. An incision was made above the fourth left rib, the periosteum stripped, and the rib resected. The pericardium was opened, and the ascending aorta cleared for implantation of a FP (20 mm, Transonic Systems Inc., Ithaca, NY, USA). The pericardium, periosteum, muscle, and skin were closed in layers. The flow probe cable was tunnelled superficially and exteriorised near the thoracic spine. Antibiotic prophylaxis (900 mg procaine penicillin, Troy Laboratories, NSW, Australia) was administered for three days after surgery. Postsurgical analgesia was maintained with intramuscular injection of flunixin meglumine (1 mg/kg) (Mavlab, Qld, Australia) at the end of surgery, then four and sixteen hours after surgery. A minimum recovery period of fourteen days was allowed prior to any study.

On the day of the study, a single experienced operator inserted a PAC-continuous cardiac output monitor (CCO) (Baxter Healthcare Corp., Irvine, CA, USA) via the right jugular vein into the pulmonary artery under 2% lignocaine local anesthesia and connected it to a Vigilance Monitor System (Baxter Healthcare Corp., Irvine, CA, USA). With the sheep lying on its right side, both the CO measures from the FP and PAC were captured from the flow meter (Transonics Systems) and the Vigilance, and recorded to computer using a CED micro 1401 interface. FP CO waveforms were acquired at a frequency of 100 Hz per beat. Ultrasound coupling gel was applied to the skin over the acoustic window between the third and fifth left ribs, and the upper thorax was insonated by a single experienced operator using a 3.3 MHz CW probe which was manipulated to optimise the transpulmonary Doppler flow profiles on the USCOM monitor. The USCOM device requires the pulmonary valve diameter (PV) to calculate flow volumes, for example, CO and stroke volume (SV). In human subjects this is determined using a proprietary anthropometric algorithm. An equivalent algorithm is not available for sheep, so for each subject, the USCOM values were calibrated to the FP measurements during a calibration phase prior to experimental measurements, with only post calibration data analysed. The USCOM investigator was blinded to the FP and PAC values, and acquired the Doppler signals and stored the flow profiles to the USCOM hard drive. Each stored screen recorded 6–12 consecutive Doppler profiles, depending on the heart rate (HR). The Doppler profiles were later traced to generate output values for each beat and stored to the USCOM hard drive for later collation and comparison with the corresponding FP and PAC measures.

### 2.2. Measurement of Outcomes

Contemporaneous CO measurements were made using all methods over a baseline period of up to 40 minutes, during i.v. infusion of the inotrope dobutamine (0.2, 0.4 and 1.0 mg/min for 40–50 min), during the postdobutamine recovery phase (10 min), during infusion of the vasopressor arginine vasopressin (AVP) (15 ng/min for 15–20 min), and during a 5 min post vasopressor phase. The order of dobutamine and AVP infusions was randomised, and a recovery period of 30 minutes between treatments was allowed for CO to return to control levels. As this study compared two beat-to-beat methods, USCOM and FP, against a time averaged method, PAC, 6 to 13 sequential measures of CO by USCOM, and FP were acquired at each comparison point on each subject, depending on heart rate, and averaged to represent the mean CO values at each time point for comparison with PAC values. Further no time to measurement after intervention was less than 4 minutes, thus allowing PAC time to respond to any CO changes.

PAC is an uncalibrated method functioning by detection of temperature gradients across a known distance so was not calibrated. However to address any potential for bias, we performed a post hoc calibration of PAC measures to baseline FP values to generate a FP-calibrated PAC series (cPAC), for comparison with FP measures.

### 2.3. Statistical Analysis

Continuous variables were expressed as mean and standard deviation (SD) and data were analysed using SPSS v.16 (SPSS Inc, Chicago, Ill, USA). Analysis included Bland Altman determination of reliability and reproducibility [39], analysis of means, standard deviations (SD), regression analysis, and Pearson's correlation coefficient. Percentage changes during and after interventions and receiver operator characteristic statistics (ROC) was calculated for PAC and USCOM compared to FP. Area under the ROC curves (AUC) were calculated for 5% incremental changes in CO between −40 and +40% CO to determine the sensitivity to change of both methods [40, 41] with acceptable sensitivity to change defined as AUC > 0.70 [42].

## 3. Results

### 3.1. Baseline Characteristics and Exclusions

A total of 363 CO measures by FP, 370 measures by USCOM and 293 PAC measures were collected from the 10 adult ewes (39 ± 4 kg). PAC failed in 20% of the experimental acquisitions, while satisfactory FP and USCOM measurements were obtained in all animals and at all time points. Statistical analysis excluded calibration measurements and included only data from contemporaneous, valid, triplicate measures, leaving 280 FP, PAC, and USCOM measures for comparison. A mean of 29 ± 11 triplicate measures were completed on each sheep (range 5−42 measures). A further 280 cPAC measures were calculated post hoc by calibrating PAC with baseline FP reference measures to complete the data set.

### 3.2. Main Outcomes

**Figure 1:**
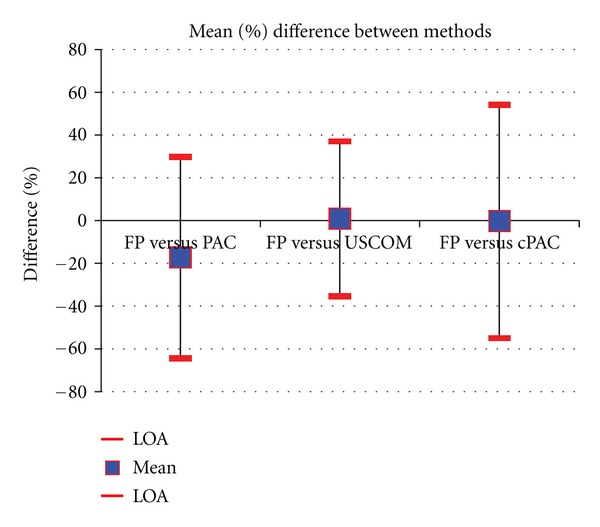
Percentage bias and precision for all paired measures for FP versus PAC (−17.2% and 47%), FP versus USCOM (1% and 36.4%), and FP versus cPAC (−0.2% and 54.4%).

**Figure 2:**
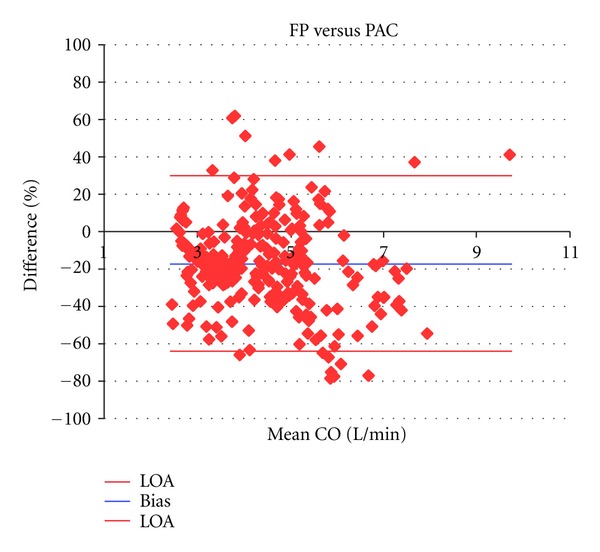
Bland Altman plots of FP versus PAC showing bias (−17.2%) and LOAs (−64.2% and 29.8%).

**Figure 3:**
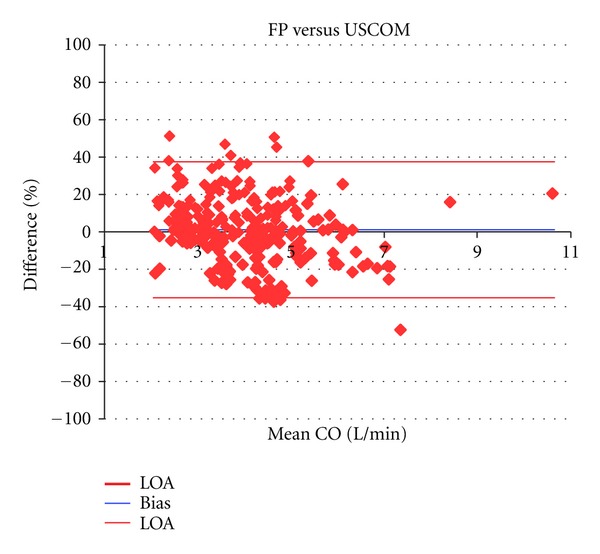
Bland Altman plots of FP versus USCOM showing bias (1%) and LOAs (−35.3% and 37.4%).

**Figure 4:**
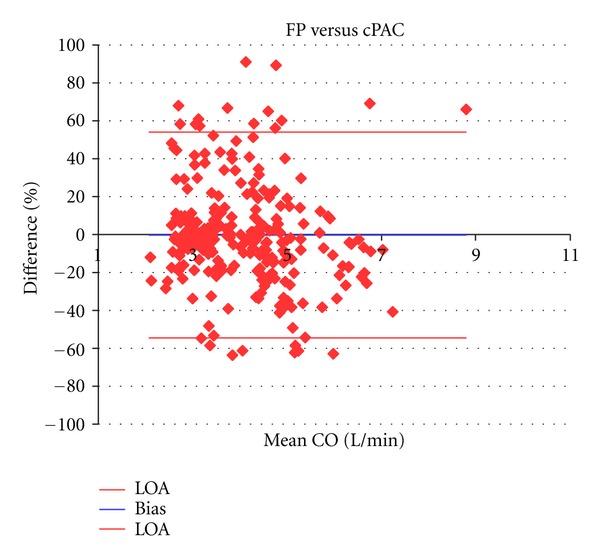
Bland Altman plots of FP versus cPAC showing bias (0.2%) and LOAs (−54.7% and 54.2%).

Mean CO by FP, PAC, USCOM and cPAC across all measures was 4.0 ± 1.2 L/min (range 1.9 to 11.7/min), 4.8 ± 1.5 L/min (range 2.5 to 10.1 L/min), 4.0 ± 1.4 L/min (range 1.7 to 9.5 L/min), and 4.1 ± 1.4 L/min (range 2.4 to 7.8 L/min), respectively (*n* = 280). CO varied across a sixfold range (1.9 to 11.7 L/min) during the experiment. The mean percentage bias and precision between paired measures for FP and PAC were −17.2% and 47.0% (Limits of agreement (LOA) −64.2 to 29.9), for FP and USCOM these were 1.0% and 36.4% (LOA −35.3 to 37.4), and FP and cPAC −0.2% and 54.4% (LOA −53.6 to 53.1) (Table 1, Figures 1, 2, 3, and 4). Calibration of PAC to the FP, cPAC, improved the bias when compared with FP from −17.2% to −0.2%, but the error in precision increased from 47.0% to 54.4%.

Regression analysis demonstrated a correlation between FP and PAC of *y* = 0.780*x* + 1.679 L/min, compared with correlations of *y* = 0.927*x* + 0.308 L/min for FP and USCOM and *y* = 0.634*x* + 1.521 L/min for FP and cPAC. Pearson correlation across all CO measurements by FP and PAC was *r* = 0.604, while for FP and USCOM it was *r* = 0.813, and for FP and cPAC *r* = 0.588.

The mean dobutamine induced CO change from baseline measured by FP was 35% (3.85 ± 0.93 to 5.13 ± 1.31 L/min) while for PAC the measured dobutamine changes were15% (4.70 ± 1.49 to 5.31 ± 1.67 L/min), an absolute under measurement of 20% compared to FP, or a relative under measurement of 56%. USCOM measured a 39% change (3.67 ± 0.96 to 5.04 ± 1.43 L/min), an absolute difference of 4%, or a relative difference of 10% from FP. For cPAC the change was 13% (3.79 ± 1.23 to 4.30 ± 1.26 L/min), a 22% absolute under measurement or a relative under measurement of 63%. The decrease in CO induced by AVP was 13.7 ± 7.35% for FP, 9.5 ± 10.5% for PAC, 5.3 ± 17.5% for USCOM, and 4 ± 13.5% for cPAC (Table 2).

**Figure 5:**
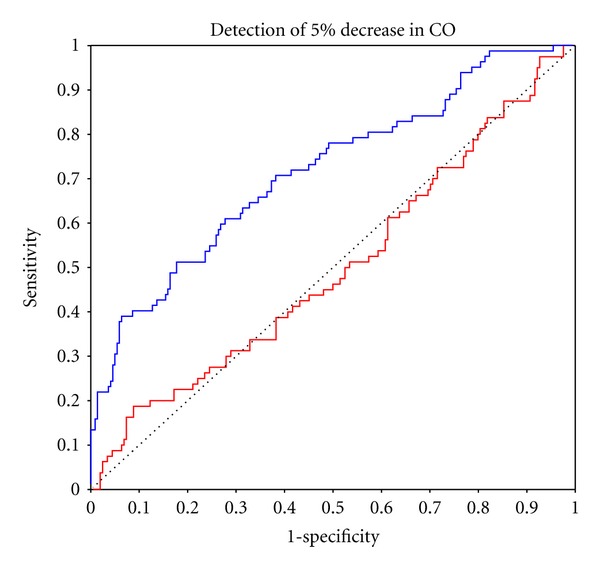
ROC curve for 70% certainty of detection of 5% decrease in CO from baseline with PAC in red (AUC = 0.496), USCOM in blue (AUC = 0.715). Random values are represented by the dotted line (AUC = 0.50) and clinical effectiveness AUC ≥ 0.70.

**Figure 6:**
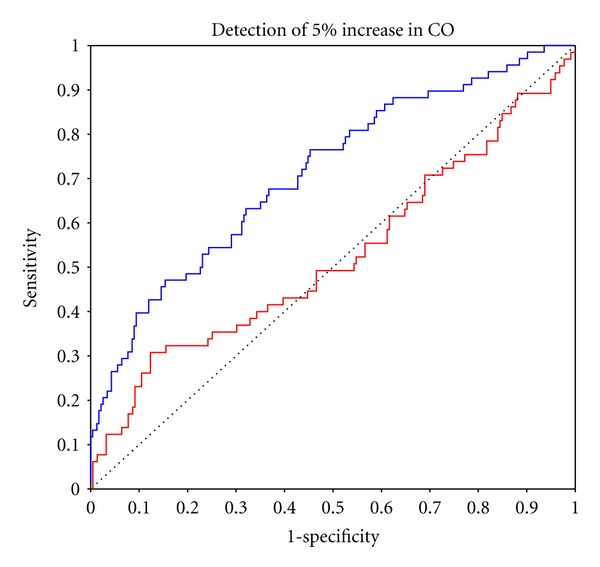
ROC curve for 70% certainty of detection of 5% increase in CO from baseline with PAC in red (AUC = 0.524), USCOM in blue (AUC = 0.708). Random values are represented by the dotted line (AUC = 0.50) and clinical effectiveness AUC ≥ 0.70.

Reliable sensitivity to CO change, ROC AUC > 0.70, was achieved by PAC for changes in excess of −30% but not +40%, with AUC values for ±5% changes being 0.524 and 0.496, respectively, indicating random values (Figures 5 and 6). USCOM reliably detected all incremental CO changes down to ±5%, where AUC values were 0.708 and 0.715. USCOM was more sensitive to CO change than PAC for all values from −40% to +40% (Table 3).

Calibration of the PAC to the FP improved the agreement but not the precision nor sensitivity of the method (Table 1).

The FP calibration of USCOM allowed calculation of sheep PV from a regression equation relating sheep weight (kg), where PV = 0.0132 weight + 1.0329 cm.

## 4. Discussion

Despite no history of validation or efficacy, PAC has been a clinical standard for hemodynamic measurement, diagnosis and monitoring, and applied as a research method and reference standard against which new CO measurement methods have been compared. This study found that PAC was an inaccurate measure of CO and was unreliable for detection of CO changes less than 30–40%. These findings may explain the absence of reported outcomes benefit associated with PAC use and raises questions as to its continued use as a gold standard hemodynamic monitor. USCOM demonstrated equivalence to FP measures across COs from 1.9 to 11.7 L/min, and reliably detected ±5% changes in CO.

Prior comparison studies generally demonstrate USCOM to have acceptable agreement with PAC but good agreement with proven CO measures such as FPs and external cardiac pumps. Prior comparisons of USCOM with PAC in postcardiac surgical critical care patients have reported bias's of 12%, 18%, and 19% [43–45], similar to the18% found in this study. While mean errors in precision reported for USCOM PAC comparisons in a variety of clinical groups averaged 30% [28–33, 37, 43–46], the recommended acceptable cutoff value [47]. However, comparisons between USCOM and non-PAC methods have demonstrated superior agreement. Critchley et al. compared USCOM with FPs in dogs and found a bias of less than 1% and an error in precision of 13% across 319 paired measures [25]. In heart failure patients USCOM CO measures demonstrated a bias of less than 1% and an error in precision of less than 10% when compared with values from external circulatory pumps driving transplanted artificial hearts [27]. The results from the current side-by-side study demonstrate that PAC compares poorly with both FP and USCOM as a measure and monitor of CO, findings consistent with the prior studies.

The Bland Altman method is the standard statistical method for comparison of two clinical methods [39], and PAC has been the preferred reference method for validation studies. However, PAC coefficient of variation (COV), an index of repeatability, is high, being on average 28% in 8 human comparison studies with USCOM [28, 30, 32, 33, 37, 43, 44, 46]. Therefore it is almost mathematically impossible for even a perfect reference method to achieve less than a 30% error of precision compared with PAC [47]. This limitation of bias and precision analysis was noted by Bland and Altman [48] and confirms that the poor reproducibility of PAC limits its performance as a reference standard for CO method comparisons. In this study FP versus USCOM errors in precision were smaller than those of FP versus PAC, 36% versus 47%, suggesting that, as the variability of FP is a constant, the increased error in precision reflects the increased variability of PAC alone. In clinical practice this poor intrinsic repeatability of PAC is acknowledged by the averaging of 3 measurements that fall within 10% of each other to achieve a clinically acceptable measure [46]. This methodological bias may reject 3 or more PAC measures which would otherwise contribute to true PAC variability and increase the underlying COV.

Measurement of circulatory change is central to the function of hemodynamic monitoring, with detection of 15% changes in CO considered to be clinically desirable [46, 49]. However, 15% sensitivity is rarely achieved by current monitoring methods, with PAC sensitivity reported to be in the order of 30% [47], similar to the 30–40% found in this study. Walker et al. found CW Doppler sensitivity in hemodynamics models to be 2.3% [24], while trans-aortic CW Doppler measured minute distance (MD), a cross-sectional area independent measure of output, has a reported sensitivity of 11% and 20% [50, 51]. In this study USCOM was found to reliably detect ±5% changes in CO 70% of the time while 15% changes were detected with 80 to 85% certainty (Table 3). This high sensitivity is predicted by Moulinier et al. [50] who identified an 11% sensitivity of a single repeated Doppler CO/SV measure. Further Moulinier demonstrated that with repeated observations this sensitivity was increased by a function of $1/\sqrt{n}$, where *n* is the number of repeated observations averaged to constitute the reference measure. The mean number of repeated observations in this study was 9 (range 6–12), meaning the predicted sensitivity of USCOM, using generalizability theory and 9 repeated measures, is $11\%\times 1/\sqrt{9}$ or 4%, a value similar to the sensitivity determined by the ROC AUC statistic of 5% found in this study. PAC, using the same AUC > 0.70 to define acceptable sensitivity, detected a −30% change in CO but not a +40% change relative to the FP (Table 3), a value broadly in line with prior studies. Boyle et al, in a PAC USCOM comparison in ICU patients, found an AUC of 67% for detection of 15% changes [43], while in a similar comparison Thom et al. found a 50% sensitivity for detecting 15% changes [44]. However, these comparisons with PAC as the reference standard prove only that one of the methods, PAC or USCOM, is insensitive to change and not which method is inaccurate. The current study suggests that the insensitivity of PAC is the source of this disagreement and not, as Boyle and Thom hypothesised, the unreliability of USCOM.

The current data also demonstrate that PAC under measured the CO change associated with inotropic intervention, a critical and common intervention in the deranged circulation, by 20% (relative 66%) compared with the FP, while USCOM differed by 4% (relative 10%). The differences between measured CO changes associated with vasopressors, where the hemodynamic changes were smaller, were less marked (Table 2). This study of normal sheep determined that the mean inotropic reserve associated with a standard weight indexed dobutamine dose was approximately 35%, and that a normal vasopressor dose reduced CO by approximately 14%.

While experimental measures of accuracy provide insight into the theoretical capability of a modality, clinical utility remains the test of effectiveness of a method, and a number of studies have been conducted to establish the clinical utility of PAC.

Connors et al. in a multicentre RCT demonstrated a relative increase in mortality of 26% and an increase in hospital costs by 38% associated with PAC use in 5,735 critically ill patients [19]. Shah et al. in a meta-analysis of 13 randomised controlled trials (RCT) between 1985 and 2005 studying PAC use in 5,051 critically ill patients found no mortality benefit or reduced in hospital stay despite an increased use of inotropes and vasodilators [2] in PAC patients. The ESCAPE trial, a 26 centre prospective RCT of PAC use in 433 acute heart failure patients was prematurely halted when the National Heart, Lung, and Blood Institute Data Safety Monitoring Board cited concerns about excess adverse events and little likelihood of a positive outcome [4]. The American Society of Anesthesiology in a literature review identified an associated mortality of 0.2 to 1.5% with PAC use, and an incidence of catheter tip infection in excess of 19% and an attributable sepsis rate of 0.7 to 3% [18].

 A commonly cited benefit of PAC is that it provides filling pressures which can be used to identify fluid responsiveness and guide fluid administration. However, these filing pressures have been found to be neither uniformly accurate [52], nor effective [2, 42, 52] for fluid guidance with Marick et al. demonstrating an AUC of 0.56 [42]. USCOM-measured SV changes with autologous physiologic challenges have been shown to detect fluid responsiveness with a positive predictive value of 91% in a patient group that included subjects in atrial fibrillation, on and off mechanical ventilation, and on vasopressors [53]. In the same study the invasively measured central venous pressure (CVP) and mean arterial pressure (MAP) were not significantly predictive of fluid responsiveness with ROC AUC values of 0.52 and 0.62, respectively. Further Sturgess et al. [49] in a study of septic subjects identified a correlation of 0.81 between USCOM measured corrected flow time (FTc) with fluid responsiveness, while CVP and brain natriuretic peptide (BNP) showed no significant correlation with fluid responsiveness, *r* = 0.4 and 0.3, respectively. Both of these studies relied on the sensitive detection of small incremental changes in flow volume (CO/SV) using USCOM to describe an individual patient's Frank Starling curve and identify fluid responsiveness, a utility which remains unproven in PAC.

The accuracy of CO/SV measures determined by USCOM is dependent on the accuracy of the prediction of the valve area and any error in this calculation will convert directly to an error in CO/SV. Two studies have reported poor agreement of transthoracic and transesophageal echocardiographic measurement of the aortic valve (AV) and pulmonary valve (PV) diameter compared with the morphometrically determined USCOM values. These studies consequently reported poor agreement of echocardiographic, and PAC determined CO values [54, 55]. USCOM's morphometrically calibrated Doppler method is based on height and weight determined AV and PV annular diameters to calculate flow volumes, with the algorithm derived from normal 2D echocardiographic data [56]. While the 2D echocardiographic measurement of valve diameters requires meticulous methodology for accurate results [57] and has significant associated errors, Capps et al. made direct measurement of the AV and PV diameters of 6801 cardiac donors using Hegar dilators and derived morphometric regression equations with values which approximate those of the USCOM algorithms [58]. Alternatively, the AV or PV diameter, measured by another method such as echocardiography or MRI, can be manually input to over ride the USCOM algorithm and preserve reliability, or a CSA-independent method such as MD, can be used to track changes and monitor central circulation changes.

The outcomes benefits of hemodynamic optimization seem intuitive and are the rationale for circulatory interventions with fluid, inotropes, and vaso-active therapies. In presurgical patients hemodynamic optimization has been demonstrated in a 29 study meta-analysis to produce a decrease in overall mortality (7.6%) and morbidity [59]. While the impact of circulatory optimization in septic shock has an even greater impact with a reported reduction in mortality of 16% (relative 34%) in adults, and 27.4% (relative 70%) in children [60, 61]. A noninvasive device which can rapidly and accurately provide physiologically rational goals such as SV and SVR for guidance of fluid, inotropes and vasoactive therapies may increase the adoption of effective hemodynamic strategies across a variety of clinical applications and take advanced hemodynamics beyond the critical care.

### 4.1. Limitations

The PAC is used for CO measurement in sheep and as a cardiovascular research tool, while the USCOM algorithms were developed in human subjects. To compensate the USCOM device was calibrated to baseline FP measures, and this may have conferred some benefit to the FP/USCOM bias comparison. However, calibration data were excluded from analysis and 76% of all measurements were made after interventions which altered CO across a sixfold range (1.9 to 11.7 L/min), thus mitigating any conferred benefits from calibration. Additionally the interventional component of the study, designed to assess sensitivity to CO change, involved calibrating all methods to a nominal zero baseline, thus removing any advantage for any method. As a further precaution a calibrated PAC series, cPAC, was generated post hoc to address any methodological bias in the study.

We found a 20% failure rate of PAC during the monitoring of normal sheep. We hypothesised that as CCO PAC has a thermometrically triggered safety cutoff, and that the normal body temperature of sheep is 39° to 39.9°C [62], higher than that of humans, 37°C [63], this may have triggered the cutoff. However, we have no explanation for this high PAC failure rate which may be a limitation of this study.

This study was of a continuous CO iteration of PAC, CCO, and so some of the observations may not be interchangeable with bolus thermodilution. However, CCO is a thermodilution method and has been adopted on the basis of very good to excellent agreement with iced bolus thermodilution [64].

The FP was implanted around the ascending aorta measuring left heart CO while both PAC and USCOM measured right CO; however, in the absence of a shunt and disregarding coronary flow, the values should equate and the comparison remain valid.

The PAC was sited in the pulmonary artery throughout the studies, and would have caused some disturbance to the flow characteristics measured by USCOM. This limitation of the catheter measurements cannot be overcome in the current preparation, and would also be a confounding factor in clinical practice.

### 4.2. Conclusion

This study found that PAC was neither accurate nor sensitive when compared with the FP, findings which may explain the apparent ineffectiveness of PAC in clinical practice. This study also found that USCOM provided equivalent CO measurements to FP and is a noninvasive and accurate alternative to PAC reliably detecting ±5% changes in CO.

## Figures and Tables

**Table 1:** Summary of comparison of methods for all paired measures and all sheep as absolute values and % values (*n* = 280).

	Mean (l/min)	Bias (l/min)	LOAs (L/min)	Bias %	Precision %	LOAs %
FP versus PAC	4.4 ± 1.3	−0.8 ± 1.3	−3.3 *to* 1.7	−17.2	47.0	−64.2 *to* 29.9
FP versus USCOM	4.0 ± 1.2	0.0 ± 0.8	−1.6 *to* 1.6	1.0	36.4	−35.3 *to* 37.4
FP versus cPAC	4.0 ± 1.1	−0.1 ± 1.2	−3.0 *to* 1.8	−0.2	54.4	−54.7 *to* 54.2

**Table 2:** Mean percentage change of CO from baseline (0%) at each intervention and recovery time-point in all sheep by each method.

% Difference	Baseline	Dobutamine	Post Dob	Vasopressor	Post Vaso
FP	0	35.4	1.2	−13.7	−13.2
PAC	0	15.7	32.5	−9.5	−16.3
USCOM	0	39.1	5.7	−5.3	−4.1
cPAC	0	13.2	30.0	−4.0	−13.1

**Table 3:** ROC area under the curve (AUC) values for detection of increased and decreased percentage changes of CO relative to FP where *P* is significance of difference between the two measures. An AUC of 1 represents perfect sensitivity, 0.7 represents clinically acceptable sensitivity to change, while 0.5 is a random relationship.

	−40%	−30%	−20%	−15%	−10%	−5%	+5%	+10%	+15%	+20%	+30%	+40%
PAC	0.855	0.811	**0.635**	**0.549**	**0.534**	**0.496**	**0.524**	**0.585**	**0.631**	**0.659**	**0.631**	**0.621**
USCOM	0.897	0.881	0.885	0.857	0.842	0.714	0.708	0.754	0.812	0.814	0.790	0.897
*P*	0.45	0.3	0.004	0.000	0.000	0.000	0.000	0.005	0.01	0.04	0.094	0.06
